# Network and Factor Structure of Depression and Anxiety Symptoms in Telemental Healthcare Patients From Bangladesh: Evidence for Precision Mental Healthcare

**DOI:** 10.1155/da/9552571

**Published:** 2026-06-17

**Authors:** Md Hafizur Rahman, Ridwana Maher Manna, Nasimul Ghani Usmani, Pradip Chandra, Md Bony Amin, Tasnu Ara, Md Robed Amin, Maruf Ahmed Khan, Helal Uddin Ahmed, Ema Akter, S. M. Hasibul Islam, Anisuddin Ahmed, Mohammad Sohel Shomik, Shams El Arifeen, Aniqa Tasnim Hossain, Ahmed Ehsanur Rahman

**Affiliations:** ^1^ Maternal and Child Health Division, International Centre for Diarrhoeal Disease Research, ICDDR, B, Mohakhali, Dhaka, 1212, Bangladesh, icddrb.org; ^2^ Institute for Population and Precision Health (IPPH), The University of Chicago, 5841 S Maryland Avenue, Chicago, 60637, Illinois, USA, uchicago.edu; ^3^ Non-Communicable Disease Control (NCDC), Director General of Health Services (DGHS), Ministry of Health and Family Welfare (MoHFW), Mohakhali, Dhaka, 1212, Bangladesh, mohfw.gov.bd; ^4^ Department of Child, Adolescent and Family Psychiatry, National Institute of Mental Health (NIMH), Sher-e-Bangla Nagar, Dhaka, 1207, Bangladesh, nimh.nih.gov; ^5^ Global Health and Migration Unit, Department of Women’s and Children’s Health, Uppsala University, 753 10 Uppsala, Sweden, uu.se

**Keywords:** anxiety, bridge symptoms, central symptoms, depression, factor analysis, network analysis, precision mental health, symptom network, telemental healthcare

## Abstract

**Background:**

Identifying core mental health symptoms is crucial for precision‐targeted interventions, especially in resource‐limited settings. However, symptom structures among individuals actively seeking telemental healthcare remain underexplored in Bangladesh and similar contexts. This study aimed to map symptom severity, factor structures, and interrelationships between depressive and anxiety symptoms to inform precision‐driven mental healthcare approaches.

**Methods:**

We conducted an observational study among 4,900 patients who attended a health facility‐based telemental healthcare in Bangladesh from January 2023 to July 2024. We assessed depression using PHQ‐9 and anxiety using GAD‐7 and applied exploratory factor analysis and network analysis.

**Results:**

Overall, 84% (95% CI: 83–86) screened positive for depressive symptoms, 85% (95% CI: 83–86) screened positive for anxiety symptoms, and 77% (95% CI: 76–78) presented co‐occurring symptoms. Commonly reported symptoms included fatigue (57%), anhedonia (42%), sleep disturbance (42%), nervousness (70%), and uncontrollable worrying (66%). Factor analysis revealed “depressed mood” (λ = 0.58) and “anhedonia” (λ = 0.51) as core depressive features, and “uncontrollable worry” (λ = 0.68) and “nervousness” (λ = 0.61) as core anxiety features. Network analysis revealed strong associations between “anhedonia” and “depressed mood” in depression and “trouble relaxing” and “restlessness” in anxiety. “Uncontrollable worrying” showed the highest centrality, and “sleep disturbance” and “trouble relaxing” served as important bridge symptoms linking depression and anxiety domains.

**Conclusions:**

Depressive and anxiety symptoms in people seeking telemental healthcare cluster around a small number of key and connecting symptoms, rather than contributing equally to overall distress. Precision mental healthcare in resource‐limited settings can use this structure to direct limited time and resources toward the symptoms that matter most.

**Protocol Registration:** Institutional Review Board (IRB) of https://www.icddrb.org/. Protocol number: PR‐22103.

## 1. Background

Mental health challenges in Bangladesh are quietly escalating amid rapid urbanization, climate stressors, and complex cultural dynamics [1, 2]. The country grapples with serious deficiencies in its mental health infrastructure, marked by a lack of public services, a dearth of qualified professionals, chronic underfunding, and deeply rooted social stigma [3]. Fewer than 50 outpatient mental health clinics serve over 170 million people in the country, most located in major cities, highlighting a severe urban–rural divide in access to mental healthcare [4]. Moreover, the country had only about 1.2 mental healthcare providers per 100,000 people, the majority concentrated in advanced care centers based in major urban areas [3, 5]. Furthermore, the COVID‐19 pandemic intensified the country’s mental health distress, laying bare the critical gaps in care and underscoring the need for widespread, accessible solutions that can scale across Bangladesh [6].

To address these pressing shortcomings, the Government of Bangladesh launched a National Mental Health Strategy aiming to embed telemental health services across all tiers of the healthcare system by 2030 [5]. Telemental health aims to enhance access to mental healthcare by connecting patients with mental health providers through a videoconferencing system [7]. Beyond specialized treatments, both the National Mental Health Strategic Plan 2030 and the World Health Organization’s (WHO) Mental Health Gap Action Programme (mhGAP) stress the importance of training frontline healthcare workers. They also highlight the need for effective communication strategies to boost public awareness and shift social attitudes around mental health [5, 7, 8].

Although clinicians often attend to presenting symptoms rather than strictly applying DSM‐based diagnostic protocols, this symptom recognition is frequently informal and not guided by structured, symptom‐targeted frameworks. At the same time, treatment decisions continue to be driven by broad diagnostic labels, which may obscure heterogeneity in symptom profiles. This combination, unsystematic symptom recognition coupled with diagnosis‐driven treatment selection, may contribute to the persistent gap between treatment need and effectiveness, with nearly 60% of patients failing to respond or relapsing within 12 months [9, 10]. Tackling this issue demands a clearer grasp of symptom patterns in patients seeking mental health services. Such insight is essential for shaping provider training, customizing communication strategies, and advancing more precise, individualized treatment approaches.

Notably, while the Bangladesh [11] National Institute of Mental Health (NIMH) reported a prevalence rate of 7% for depressive symptoms and 5% for anxiety symptoms, these figures lack information on the symptoms of depression and anxiety among people seeking primary mental healthcare. Additionally, national figures reflect the general population and may underestimate the symptom burden among individuals actively seeking telemental healthcare. Other studies also explored depression among specific outpatient groups, such as elderly outpatients [12], those with gastrointestinal symptoms [13], pregnant women [14], and those with type‐2 diabetes in Bangladesh [15, 16]. However, prior studies did not explore the symptoms of depression and anxiety among people seeking mental healthcare. This gap hinders the efficacy of facility‐based psychological interventions by leaving uncertainty about which symptoms to prioritize and which therapy methods to adopt.

A wide range of assessment tools is used to evaluate mental health issues, particularly depression and anxiety. However, these tools often differ in the symptoms they emphasize and create inconsistencies that can complicate diagnosis and treatment [17]. Prior knowledge of the specific symptoms most indicative of each condition can significantly enhance a psychologist’s ability to accurately identify patients’ core issues and prioritize the most pressing concerns. Such a targeted approach not only improves diagnostic precision but also supports the development of tailored treatment plans, particularly in telemental health care settings, where the nuances of mental health can be harder to detect without in‐person interaction [17, 18]. Moreover, growing evidence suggests that understanding symptom dimensions, rather than relying solely on categorical DSM diagnoses, offers deeper insights into patients’ overall functioning and enhances the effectiveness of personalized interventions [19, 20]. Identifying patterns in symptom presentation is, therefore, critical for advancing precision‐driven therapeutic strategies. Despite the importance of understanding symptom patterns among telemental healthcare users, no research has systematically examined the symptom structures and interrelationships within this population. Thus, this study aimed to map symptom severity, factor structures, and interrelationships of depressive and anxiety symptoms to inform precision‐driven approaches.

## 2. Methods

### 2.1. Study Context and Participants

In collaboration with the NIMH, the International Centre for Diarrhoeal Disease Research, Bangladesh (ICDDR,B), guided by the Bangladesh government’s Non‐communicable Disease Control (NCDC) program, has launched a pilot initiative known as the Wellbeing Center. This telemental health platform delivers entry‐level psychological support and psychiatric services via videoconferencing, facilitated by trained clinical and educational counselors at four public health sites across Bangladesh [7]. Facilities are Dinajpur District Hospital (DH), Netrokona DH, Chirirbandar Upazila Health Complex (UHC), and Durgapur UHC. Among these, Dinajpur DH and Netrokona DH provide secondary‐level healthcare, and Chirirbandar UHC, and Durgapur UHC provide primary‐level healthcare. A map with the location of the facilities is given in the Figure S1. Data were collected from patients aged 10 and older who completed a telemental healthcare consultation.

### 2.2. Data Collection Procedure

Established in September 2022, the Wellbeing Center began collecting data in January 2023. Initially, doctors and healthcare providers across various departments such as medicine, emergency medicine, cardiology, health education, orthopedics, gynecology, and obstetrics, and noncommunicable diseases, or from neighboring healthcare facilities, identify patients with mental disorders using the mhGAP tool [8] and refer them to the Wellbeing Center for telemental healthcare. Additionally, patients also walk in directly to the Wellbeing Center. When a patient arrives at the Wellbeing Center, a field assistant—trained through an intensive 3‐week programe—conducts the initial intake. This includes administering the PHQ‐9 and GAD‐7 to screen for depression and anxiety, registering the patient into the Electronic Health Record (EHR) system via tablet, and completing all steps before the counseling session. Details of the patient flow and data collection points are provided in the Figure S2. Data cleaning involved removing incomplete, duplicate, or inconsistent responses, with regular weekly supervision by three field research supervisors. Issues or inconsistencies were resolved by contacting field assistants.

### 2.3. Study Design and Sample

A cross‐sectional analysis was carried out using patient mental health data from EHR records, including PHQ‐9 and GAD‐7 scores and covariates from patient registration, collected during patients’ first visits to four Wellbeing Centers between January 2023 and July 2024. Of the 5028 patients approached, 4,987 participated in baseline screening. Analyses were conducted as a complete‐case analysis, including only participants with complete PHQ‐9 and GAD‐7 data at the initial telemental healthcare visit; treatment completion and follow‐up outcomes were not assessed. For descriptive analyses, missing values in sociodemographic variables, including education, household income, and referral source, were retained and reported as an “Unknown.” Because missingness in covariates was minimal (<0.5% for all variables), inferential analyses are reported using the full analytic sample size (*N* = 4900), with observations containing missing covariate values excluded pairwise as required.

Outliers in continuous variables, including age, education, and income, were identified using the interquartile range (IQR) method, defined as values below Q1–1.5 × IQR or above Q3 + 1.5 × IQR, and were excluded. After removing outliers of continuous variables, children under 10 years, and cases with missing values for outcome variables, 4900 participants were included in the final analysis.

### 2.4. Data Collection Tools, Variables, and Categorization

The EHR system recorded patients’ mental health data using a structured questionnaire Table S1. Both PHQ‐9 and GAD‐7 use a 4‐point Likert scale with response anchors: 0 (“Not at all”), 1 (“Several days”), 2 (“More than half the days”), and 3 (“Nearly every day”), assessing symptom frequency over the past 2 weeks.

We assessed depression using the PHQ‐9, which evaluates nine core symptoms: reduced interest or enjoyment in activities, persistent sadness, sleep disturbance, fatigue, appetite changes, feelings of low self‐worth, trouble focusing, slowed physical or mental activity, and thoughts of self‐harm [21]. The instrument has been adapted for the Bengali language, validated for local use (Cronbach’s α = 0.837), and is frequently employed in mental health studies in Bangladesh [2, 14, 22].

The GAD‐7 scale, assessing anxiety, covered symptoms including feeling on edge, inability to manage worrying, excessive concern, difficulty relaxing, restlessness, irritability, and anticipating negative events [23]. This tool has been translated and culturally adapted for Bangladesh, with strong reliability in validation studies (Cronbach’s α = 0.869) [2, 24].

Depression levels based on PHQ‐9 were stratified into five tiers: none/minimal (0–4), mild (5–9), moderate (10–14), moderately severe (15–19), and severe (20–27). For GAD‐7, anxiety symptoms were similarly tiered into none/minimal (0–4), mild (5–9), moderate (10–14), and severe [14, 23, 25]. Consistent with regional validation and prior Bangladeshi studies, participants scoring ≥10 on the PHQ‐9 or GAD‐7 were classified as having depression or anxiety, respectively [2, 14, 26]. If both conditions were met, the case was marked as comorbid and treated as a binary indicator in the dataset [14].

The dataset also included sociodemographic and economic attributes: respondent age, sex, relationship status, type of work, education (in years), and monthly income in Bangladeshi Taka. Initially collected as continuous fields, age, education, and income were later categorized for analysis to improve interpretability and facilitate comparison across clinically and programmatically meaningful groups. Age brackets were defined as adolescents (10–19 years), young adults (20–39), middle‐aged adults (40–59), and older adults (60+). Gender responses were limited to male or female; no other identities were recorded. Consistent with another study, participants were further segmented by care level; those who visited DHs were classified as district‐level patients, while those who visited UHCs were categorized as sub‐district level [14].

### 2.5. Statistical Analysis

All analyses were conducted using *R* version 4.3.1. We used descriptive methods, including means, frequencies, and percentages, to summarize the data. Associations between depression and anxiety symptoms and key variables were tested using chi‐square statistics. Prevalence of mental distress was presented with 95% confidence intervals.

We conducted two independent exploratory factor analyses (EFAs): one using the nine PHQ‐9 items and one using the seven GAD‐7 items. These EFAs were conducted within each scale separately to characterize latent symptom structure, whereas network analysis was estimated on the combined 16 PHQ‐9 and GAD‐7 items to examine cross‐scale partial correlations and identify bridge symptoms between depression and anxiety domains. EFAs were used to examine item‐level symptom structure and relative contributions within each scale in this telemental healthcare population, rather than to formally test a predefined measurement model. To assess consistency across subgroups, EFAs were re‐estimated separately by sex and age group; factor structures and loading patterns were highly similar across subgroups. To assess data suitability for factor analysis, the Kaiser–Meyer–Olkin (KMO) test was applied, with a threshold of 0.60 or higher considered acceptable. Sampling adequacy was excellent for both EFAs (KMO > 0.80 for PHQ‐9 and GAD‐7). Bartlett’s test of sphericity was statistically significant for both EFAs (PHQ‐9: df = 36, *p*  < 0.001; GAD‐7: df = 21, *p*  < 0.001), confirming factorability of each item set. We performed exploratory factor analysis (EFA) using principal axis factoring with oblimin rotation. Items with factor loadings of 0.40 or above were retained, and no items exhibited problematic cross‐loading.

To explore direct inter‐item relationships among depressive and anxiety symptoms, we conducted network analysis using the EBICglasso algorithm via the bootnet package, which constructs sparse partial correlation networks. Polychoric correlations were calculated using the psych package, and the network was estimated via the graphical lasso method from the huge package. We evaluated the robustness of the network structure using case‐dropping bootstrapping via the *bootnet* package, computing central stability (CS) coefficients for key metrics including strength, closeness, and betweenness. A CS‐coefficient threshold of ≥ 0.50 was considered indicative of acceptable stability [27], and results confirmed the robustness of the network metrics across various subsamples. Expected influence (EI) was selected as the primary centrality metric because it incorporates both positive and negative edge weights and demonstrated acceptable stability in case‐dropping analyses; network matrix values, strength, closeness, and betweenness were also estimated and are reported in the Table S5–S7.

EFA identified latent symptom clusters underlying depression and anxiety, while network analysis mapped associations between individual symptoms. Together, these complementary methods provided a comprehensive understanding of symptom structures and pinpointed the most central symptoms for precision‐targeted intervention.

## 3. Results

Table 1 shows the demographic characteristics of respondents. Most of the patients were aged between 20 and 39 years, females, Muslim and married, homemakers, had 6–10 years of education, and were in the lower‐middle‐income bracket. Respondents were nearly evenly distributed between district and sub‐district areas.

**Table 1:** Respondents’ demographic characteristics (*n* = 4900)

Characteristics of the participants	*N*	%
Age (years)		
10–19	803	16.4
20–39	2702	55.1
40–59	1047	21.4
60–90	348	7.1
Gender		
Male	1207	24.6
Female	3693	75.4
Religion		
Muslim	4508	92
Others^1^	392	8
Marital status		
Married	3765	76.8
Unmarried	883	18
Divorced/widowed	252	5.1
Profession		
Involved in income generating activity^2^	871	17.8
Homemaker	3074	62.7
Student	751	15.3
Unemployed	204	4.2
Education (years)		
0–5	1790	36.5
6–10	1828	37.3
11–18	1280	26.1
Unknown	2	0.0
Household income (BDT per month)		
Very low (<10,000)	703	14.4
Lower middle (10,000–19,999)	2762	56.4
Middle (20,000–29,999)	842	17.2
High (30,000–150,000)	577	11.8
Unknown	16	0.3
Patient is from		
Referred by hospital indoor and outdoor^3^	2291	46.8
Referred by other hospitals^4^	63	1.3
Walk‐in	128	2.6
Unknown	2418	49.3
Region		
Sub‐district	2363	48.2
District	2537	51.8

^1^Hindu, Christian

^2^Job, business, farmer, mason, or daily labor.

^3^Gynecology, medicine indoor, medicine outdoor, or emergency.

^4^Community clinic, other hospital.

Table 2 presents the depression and anxiety status across demographic characteristics of respondents. In total, 84% (95% CI: 83–86) screened positive for depressive symptoms, while 85% (95% CI: 83–86) screened positive for anxiety symptoms. Higher rates of depressive symptoms were observed among middle‐aged and older adults, females, divorced or widowed individuals, unemployed participants, those with lower education levels, lower household incomes, and walk‐in patients. Similarly, participants who were middle‐aged or older, divorced or widowed, unemployed, had less education, had very low or lower‐middle household income, and were walk‐in patients had more anxiety symptoms. Distribution of depression and anxiety severity across respondent demographics and chi‐square analysis is given in the Table S3. The results of the multivariate logistic regression analysis for the presence of depressive and anxiety symptoms, along with their associated factors, are provided in the Table S4.

**Table 2:** Distribution of depression and anxiety prevalence by respondents’ demographic characteristics (*n* = 4900).

Characteristic	Depression status			Anxiety status		
	Depressive	Not depressive	*p*‐Value	Anxiety	Not anxiety	*p*‐Value
Overall	4116 (84%)	784 (16%)	—	4165 (85%)	735 (15%)	—
Age (years)						
10–19	669 (83%)	134 (17%)	0.005	631 (79%)	172 (21%)	<0.001
20–39	2254 (83%)	448 (17%)		2277 (84%)	425 (16%)	
40–59	910 (87%)	137 (13%)		931 (89%)	116 (11%)	
60–90	309 (89%)	39 (11%)		308 (88%)	40 (12%)	
Gender						
Male	989 (82%)	218 (18%)	0.004	1003 (83%)	204 (17%)	0.089
Female	3153 (85%)	540 (15%)		3144 (85%)	549 (15%)	
Religion						
Muslim	3803 (84%)	705 (16%)	0.3	3815 (85%)	693 (15%)	>0.9
Others^1^	339 (87%)	53 (13%)		332 (85%)	60 (15%)	
Marital status						
Married	3163 (84%)	602 (16%)	<0.001	3204 (85%)	561 (15%)	<0.001
Unmarried	738 (84%)	145 (16%)		707 (80%)	176 (20%)	
Divorced/widowed	241 (96%)	11 (4%)		236 (94%)	16 (6%)	
Profession						
Involved in income generating activity^2^	716 (82%)	155 (18%)	0.021	743 (85%)	128 (15%)	0.002
Homemaker	2618 (85%)	456 (15%)		2625 (85%)	449 (15%)	
Student	625 (83%)	126 (17%)		601 (80%)	150 (20%)	
Unemployed	183 (90%)	21 (10%)		178 (87%)	26 (13%)	
Education (years)						
0–5	1542 (86%)	248 (14%)	0.004	1534 (86%)	256 (14%)	0.043
6–10	1551 (85%)	277 (15%)		1555 (85%)	273 (15%)	
11–18	1047 (82%)	233 (18%)		1056 (83%)	224 (18%)	
Household income (BDT per month)						
Very low (<10,000)	600 (85%)	103 (15%)	<0.001	603 (86%)	100 (14%)	0.010
Lower middle (10,000–19,999)	2382 (86%)	380 (14%)		2363 (86%)	399 (14%)	
Middle (20,000–29,999)	697 (83%)	145 (17%)		702 (83%)	140 (17%)	
High (30,000–150,000)	450 (78%)	127 (22%)		464 (80%)	113 (20%)	
Patient is from						
Referred by hospital indoor and outdoor^3^	2002 (87%)	289 (13%)	<0.001	2020 (88%)	271 (12%)	0.021
Referred by other hospitals^4^	56 (89%)	7 (11%)		59 (94%)	4 (6%)	
Walk‐in	126 (98%)	2 (2%)		122 (95%)	6 (5%)	
Region						
Sub‐district	2008 (85%)	355 (15%)	0.4	2005 (85%)	358 (15%)	0.70
District	2134 (84%)	403 (16%)		2142 (84%)	395 (16%)	

^1^Hindu, Christian.

^2^Job, business, farmer, mason, or daily labor.

^3^Gynecology, medicine indoor, medicine outdoor, or emergency.

^4^Community clinics, other hospitals.

**Figure 1:**
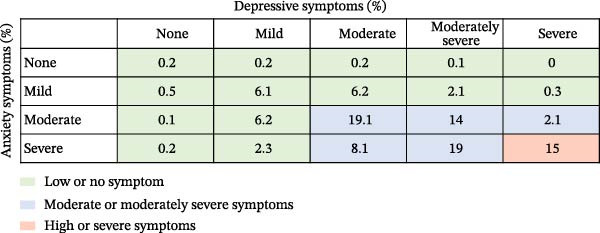
Percentage overlap of depression and anxiety co‐occurrence across varying severity levels (*n* = 4900).

Figure 1 depicts the overlap in co‐occurring depression and anxiety across varying severity levels. In total, 77% (95% CI: 76–78) of participants screened positive for both depressive and anxiety symptoms, with ~15% experiencing severe forms of both simultaneously.

**Figure 2:**
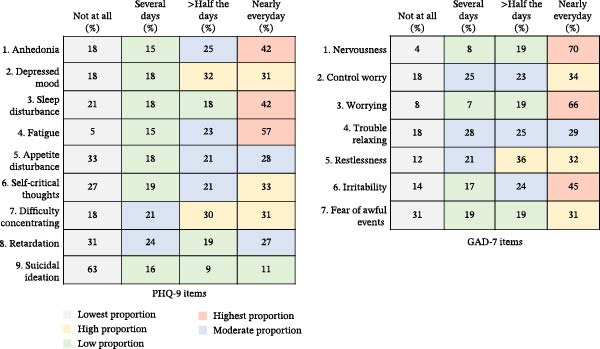
Percentage‐based heatmap of responses to individual PHQ‐9 and GAD‐7 items (*n* = 4900).

Figure 2 presents the item‐level response rates for depressive symptoms (PHQ‐9) and anxiety symptoms (GAD‐7). According to the PHQ‐9, 57% of respondents frequently experienced fatigue, while 42% reported either loss of interest or sleep issues nearly every day. GAD‐7 responses show that 70% often felt nervous or on edge, 66% expressed excessive worry, and 45% experienced frequent irritability.

**Figure 3:**
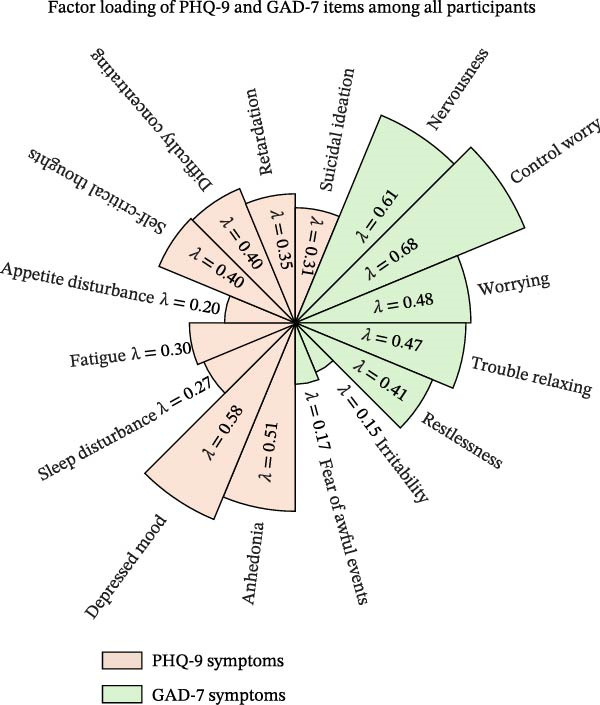
Factor loading of GAD‐7 and PHQ‐9 items among all participants (*n* = 4900).

Figure 3 illustrates the factor loadings (λ) of the GAD‐7 and PHQ‐9 items. For the PHQ‐9, “depressed mood” (λ = 0.58) and “anhedonia” (λ = 0.51) exhibit the highest factor loadings, highlighting their central role in the depression construct. Similarly, for the GAD‐7, “control worry” (λ = 0.68) and “nervousness” (λ = 0.61) show the strongest loadings. Subgroup EFAs by sex and age showed no meaningful differences in factor structure or item loading patterns (Figures S4 and S5).

**Figure 4:**
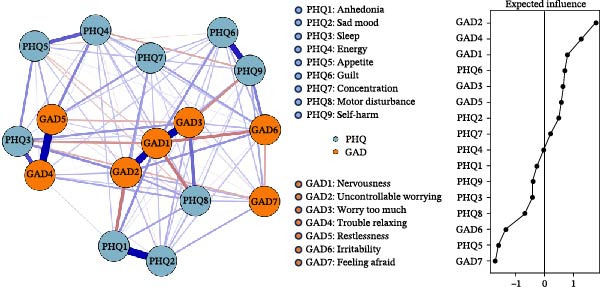
Symptom network of depression (PHQ) and anxiety (GAD) among 4,900 participants (*n* = 4900).

Figure 4 visualizes the symptom network for depression (PHQ‐9) and anxiety (GAD‐7), estimated using the EBICglasso model. Edge thickness reflects the strength of associations between symptoms. Within the PHQ‐9 cluster, the strongest connection was between PHQ1 (“Loss of interest”) and PHQ2 (“Low mood”), followed by a strong partial correlation between PHQ6 (‘Feelings of guilt’) and PHQ‐9 (‘Thoughts of self‐harm’). A moderate relationship was observed between PHQ4 (“Fatigue”) and PHQ5 (“Appetite changes”). In the GAD‐7 cluster, the most robust edge‐connected are GAD4 (“Difficulty relaxing”) and GAD5 (“Restlessness”), with additional strong links between GAD1 (“Nervousness”), GAD2 (“Inability to control worry”), and GAD3 (“Excessive worry”).

The network also highlights intercommunity connections. Notably, PHQ3 (“Sleep disturbance”) displayed strong associations with GAD4 (“Trouble relaxing”). PHQ4 (“Fatigue/energy”) and GAD5 (“Restlessness”) were also interconnected, indicating a link between fatigue levels and restlessness. Additionally, PHQ8 (“Motor disturbances”) showed connections with GAD3 (“Worry too much”) and GAD1 (“Nervousness”).

In terms of EI, GAD2 (“Uncontrollable worrying”) exhibited the highest EI centrality in the network, followed by GAD4 (“Trouble relaxing”) and GAD1 (“Nervousness”). Conversely, symptoms such as PHQ5 (“Appetite”) and GAD‐7 (“Fear of awful event/feeling afraid”) showed lower EI centrality. Strength and closeness centrality showed stability comparable to EI, whereas betweenness exhibited lower stability (CS = 0.283); corresponding values and distributions for all centrality metrics are provided in Tables S5–S7.

**Figure 5:**
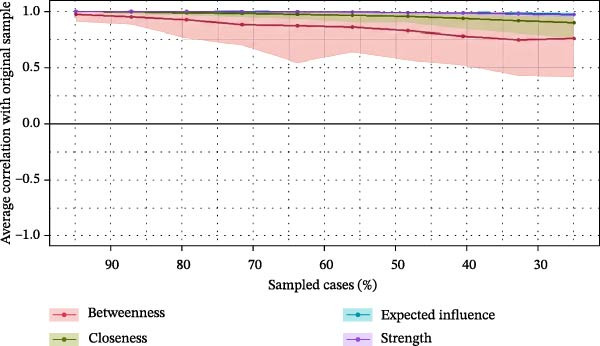
Assessment of the network stability (*n* = 4900).

Figure 5 presents the stability analysis of the symptom network. CS coefficients for closeness, strength, and EI were each 0.75, well above the 0.50 threshold, indicating strong reliability in these centrality metrics. Betweenness centrality exhibited lower stability than strength, closeness, and EI as case‐dropping increased. Formal calculation of the CS coefficient based on these results yielded a value of 0.283 for betweenness, compared with 0.75 for strength, closeness, and EI (Table S7), indicating limited robustness of betweenness estimates despite visually moderate correlations at higher sampling levels.

## 4. Discussion

This study fills critical gaps in the understanding of symptom structures among individuals seeking psychological support and psychiatric services in telemental healthcare in Bangladesh and offers robust insights for precision‐driven intervention strategies. Our findings reveal a high proportion of depressive and anxiety symptoms. The elevated prevalence of depression and anxiety symptoms in our sample likely reflects the nature of the population, individuals actively seeking or requiring primary mental health services. Comparatively, in various outpatient studies conducted in Bangladesh, the prevalence rates of depression have varied widely depending on the patient groups. Notably, these studies have predominantly focused on patients with physical health conditions such as gastrointestinal symptoms [13], pregnant women [14], and type‐2 diabetes in Bangladesh [15, 16]. Particularly concerning is the high prevalence of anxiety symptoms, an area relatively underexplored in previous outpatient studies. We also found that older adults and women exhibited greater symptom burden, consistent with global evidence [28, 29], suggesting that demographic patterns of vulnerability are similar across different sociocultural contexts.

Our findings highlight substantial co‐occurrence between depression and anxiety symptoms, underscoring the deeply interconnected and complex relationship between these conditions. Prior research suggests that this overlap reflects shared clinical features as well as common neurobiological mechanisms linking anxiety and depression [30, 31]. Such co‐occurrence may arise from overlapping neurobiological and psychosocial pathways, including neurotransmitter dysregulation, genetic susceptibility, and environmental stressors. Together, these shared mechanisms support the view that anxiety and depression may represent different expressions of a partially shared underlying psychopathological process [30, 32].

The gradation of depression and anxiety severity observed in our study, from mild to severe, aligns with the staged framework for common mental disorders put forward by The Lancet World Psychiatry Commission [33]. It advocates particular interventions corresponding to the severity of symptoms, and telemental healthcare emerges as a promising avenue. A growing body of evidence consistently supports the efficacy of telemental health services in managing mild to moderate symptoms of depression and anxiety [7, 34], leading to their increasing adoption by different healthcare systems. However, patients with severe depressive and anxiety symptoms may need a more intensive treatment approach, potentially including a combination of medication, in‐depth psychotherapy, and possibly inpatient care or specialized outpatient services, to effectively address their complex and acute mental health needs.

Both factor and network analyses in this study converged to identify specific symptoms that shape the structure and dynamics of depression and anxiety. Within the anxiety domain, the centrality of “uncontrollable worry” and “nervousness” on the GAD‐7 aligns with prior findings, underscoring their role as core features of generalized anxiety disorder and key drivers of symptom severity and chronicity [23, 35, 36]. Additionally, therapeutic approaches like cognitive behavioral therapy (CBT) and acceptance and commitment therapy (ACT) have proven effective in targeting the cognitive dimension of anxiety, especially persistent worry, which plays a major role in impairing day‐to‐day functioning [36–38]. Understanding anxiety symptoms, particularly the “worry,” can help in adopting targeted approaches, leading to more personalized and effective treatment plans, a growing necessity in mental healthcare, especially with the increasing adoption of telehealth services [36].

Similarly, in the domain of depression, the prominence of “depressed mood” and “anhedonia” in the PHQ‐9 underscores their central role in the depression construct. This pattern was consistently observed across male and female patients and all age groups. Studies have shown that these symptoms are not only prevalent but are significantly predictive of major depressive disorder (MDD) outcomes [39]. Importantly, the presence of distress and anhedonia has been associated with longer times to remission and recovery, and it highlights the need for more attention among this population [40]. While CBT remains effective for many mental disorders, it may be less efficacious for treating core depressive symptoms such as depressed mood and anhedonia [40, 41]. Early identification of patients experiencing these symptoms, combined with interventions such as antidepressants, behavioral activation, and augmented depression therapy, may yield better outcomes [40, 42].

The network analysis revealed strong interconnections between depressive and anxiety symptoms. Within depression, anhedonia and depressed mood were closely linked, highlighting how diminished positive affect is intertwined with persistent sadness, consistent with the dual‐factor model of mental health [43]. In the anxiety domain, trouble relaxing and restlessness emerged as strongly associated symptoms. Additionally, uncontrollable worrying demonstrated the highest centrality, indicating a structurally influential position within the symptom network. From a network perspective, such symptoms represent potential intervention targets; however, it remains an open empirical question whether directly targeting central symptoms leads to broader symptom improvement. Cross‐disorder associations were also evident, with sleep disturbance strongly linked to trouble relaxing, indicating that dysregulation of sleep–relaxation mechanisms may serve as a common pathway reinforcing both depressive and anxiety symptoms. Addressing these symptoms through targeted interventions, such as sleep hygiene strategies, relaxation training, and behavioral activation, may help disrupt the shared pathways of emotional distress and accelerate recovery across both disorders [44]. CBT protocols focusing on relaxation training, stress reduction, and behavioral activation could be particularly effective. Online delivery of such interventions may also enhance accessibility within telemental healthcare frameworks, addressing overlapping symptom pathways and improving overall treatment outcomes [44–46].

Using two complementary analytical approaches—factor analysis and symptom network analysis—we observed that the same symptoms, including depressed mood, anhedonia, uncontrollable worry, and nervousness, were prominent across both frameworks. Given that symptoms with high loadings on latent factors are also expected to show strong associations with other symptoms within the same scale, this convergence should not be interpreted as independent validation. Rather, the findings provide a consistent descriptive characterization of symptom prominence across related analytic perspectives. Bridge symptoms such as sleep disturbance and trouble relaxing further highlighted symptom overlap between depression and anxiety. This convergence indicates that these symptoms occupy structurally prominent positions within the cross‐sectional symptom network. However, these findings should not be interpreted as evidence of causal influence, temporal precedence, or within‐person dynamics, which require longitudinal and experimental investigation. This view aligns with contemporary models of psychopathology that conceptualize mental disorders as dynamic systems of mutually reinforcing symptoms rather than static diagnostic categories [47].

Our results align with global network analyses that consistently pinpoint low mood, loss of interest, persistent worry, and nervousness as core nodes driving both depressive and anxiety symptom networks [48–50]. This consistency across studies suggests that these symptom relationships may represent universal features of emotional disorders across different cultural contexts. However, cultural factors such as stigma, health‐seeking behaviors, and local stressors may still shape how symptoms are expressed and reported and highlight the need for future cross‐cultural symptom network comparisons to refine precision mental health strategies.

For providers, particularly in telemental healthcare settings where clinical impressions often rely heavily on patient self‐report. Interventions that focus on depressed mood, anhedonia, uncontrollable worry, and nervousness, along with bridge symptoms such as sleep disturbance and trouble relaxing, may represent promising targets suggested by symptom network structure, though their effectiveness in producing broader symptom change requires confirmation through longitudinal and experimental studies. In resource‐limited settings such as Bangladesh, this focus offers a cost‐effective strategy to maximize clinical impact, allowing providers to prioritize interventions where they are most likely to create cascading improvements across the broader symptom network. Training programs that equip providers to recognize and address these high‐impact symptoms are essential to enhancing the precision and effectiveness of care delivery. In parallel, social and behavioral change communication strategies that raise awareness about these specific symptoms can promote early help‐seeking behaviors and reduce stigma associated with mental health conditions. Therapeutic approaches that integrate symptom‐specific interventions, such as cognitive–behavioral techniques targeting worry and behavioral activation addressing anhedonia, offer additional pathways to improve patient outcomes. When two distinct analytical lenses converge on the same symptoms, they illuminate not only the heart of psychopathology but also the most promising entry points for recovery, a lesson with profound implications for the future of precision‐driven mental healthcare.

Our study has some limitations. The selection bias arising from including only clients who visited the WBC outpatient telemental health unit may limit the generalizability of the findings to the broader population. Because this study used a complete‐case analysis of baseline assessments from patients with complete symptom data, findings may not be generalizable to individuals who did not complete screening or who disengaged early from telemental healthcare services. Additionally, individuals without digital literacy or internet access were excluded, further restricting the representativeness of the sample and potentially introducing bias.

The cross‐sectional design of the study limits the ability to infer causality between the observed factors and mental health outcomes and causal interpretations of symptom relationships. Because the network was estimated using cross‐sectional partial correlations, the direction, temporal ordering, and causal validity of symptom relationships cannot be determined. Reliance on interviewer‐administered self‐reported screening tools (PHQ‐9 and GAD‐7) rather than clinician‐administered diagnostic interviews could introduce misclassification bias, affecting the precision of symptom identification. Additionally, this study focused on internal symptom structures and did not examine associations with clinically significant outcomes such as suicidality. The study focused exclusively on MDD and generalized anxiety disorder symptoms, which may not fully capture the broader spectrum of mental health issues present in outpatient populations. The facility‐based nature of the telemental healthcare service may limit the applicability of the findings to other types of mental health settings. Sixth, the lack of cultural data and detailed diagnostic information constrains exploration of cultural influences on symptom patterns and diagnostic heterogeneity. Finally, the absence of longitudinal data prevents assessment of symptom progression or causal relationships over time. Future research should address these limitations by incorporating more diverse recruitment strategies, clinician‐administered diagnostic assessments, cultural assessments, and longitudinal designs to strengthen generalizability and clinical relevance.

## 5. Conclusion

This study reports the complex interplay between depressive and anxiety symptoms among patients seeking outpatient telemental healthcare in Bangladesh. Using both factor and network analyses, we identified “depressed mood,” “anhedonia,” “nervousness,” and “uncontrollable worry” as the most influential symptoms within and across diagnostic categories. Key intercommunity bridges, such as the links between sleep disturbance and trouble relaxing, further emphasized the overlapping nature of these disorders. Notably, uncontrollable worry emerged as the most central symptom, followed by trouble relaxing and nervousness.

Rather than endorsing a one‐size‐fits‐all model, our findings emphasize the importance of precision‐driven, symptom‐focused strategies within telemental health care systems. Targeting the core symptoms of depressed mood, anhedonia, uncontrollable worry, and nervousness, along with the bridge symptoms of sleep disturbance and trouble relaxing, may help reduce the severity of depression and anxiety, weaken the interconnections between disorders, and support the delivery of precision interventions in telemental healthcare settings. In addition to therapeutic innovations, provider training programs that focus on recognizing and addressing these core symptoms, alongside social and behavioral change communication strategies to promote help‐seeking and reduce stigma, are critical to improving clinical effectiveness.

Building on these insights, future initiatives should prioritize the creation of symptom‐specific digital therapeutic modules, such as worry management tools, behavioral activation exercises, and sleep hygiene interventions. Embedding these strategies into Bangladesh’s National Mental Health Strategy 2030 could optimize the scalability and impact of telemental health services. Furthermore, our findings offer actionable guidance for mental health policy development, suggesting that resource allocation and clinical practices should focus on central symptoms to maximize treatment impact within health facility‐based systems.

Future research should move beyond static assessments of symptomatology and embrace dynamic, real‐time methodologies. Longitudinal studies using ecological momentary assessment (EMA) and digital phenotyping could illuminate how symptoms such as uncontrollable worry and anhedonia fluctuate over time, interact with environmental factors, and respond to targeted interventions. Experimental designs that selectively intervene on central symptoms may offer critical insights into whether modifying these keystone symptoms leads to broader symptom network collapse and sustained clinical remission.

## Author Contributions

Md Hafizur Rahman and Ahmed Ehsanur Rahman conceptualized and designed the study. Md Hafizur Rahman drafted the manuscript and addressed the reviewers’ comments. Md Hafizur Rahman, Nasimul Ghani Usmani, Pradip Chandra, and Md Bony Amin analyzed the data, with assistance from Tasnu Ara and Ridwana Maher Manna. S. M. Hasibul Islam, and Ema Akter facilitated data collection. Md Robed Amin, Maruf Ahmed Khan, and Helal Uddin Ahmed supervised the conduct of the study. Aniqa Tasnim Hossain, Mohammad Sohel Shomik, Shams El Arifeen, and Anisuddin Ahmed reviewed the manuscript. Aniqa Tasnim Hossain and Ahmed Ehsanur Rahman played the role of senior authors, overseeing the conduct of the study, the analysis, and the manuscript drafting process.

## Acknowledgments

The authors extend their gratitude to the NCDC division of the Ministry of Health and Family Welfare (MoHFW), Bangladesh, and the National Institute of Mental Health (NIMH) for providing invaluable support for conducting this study. Additionally, the authors express their sincere thanks to the Civil Surgeons, Superintendents, Upazila Health and Family Welfare Officers, Resident Medical Officers, and Medical Officers for their unwavering assistance throughout the study in their respective facilities. Lastly, the authors are profoundly grateful to the study participants whose involvement made this research possible.

## Funding

The study was funded by the Non‐communicable Disease Control (NCDC) division of the Ministry of Health and Family Welfare (MoHFW) of the Bangladesh Government (Grants GR‐02525, GR‐02393, and GR‐02226), This study was also funded by the Department of Foreign Affairs, Trade and Development (DFATD), Canada through Advancing Sexual and Reproduction Health and Rights (AdSEARCH) (Grant SGDE‐EDRMS‐#9926532), Purchase Order 7428855, Project P007358. Additionally, this research study conducted by ICDDR,B, receives funding from core donors, including the Governments of Bangladesh and Canada, who provide unrestricted support to ICDDR,B for its operations and research. We gratefully acknowledge our core donors for their support and commitment to ICDDR,B research efforts.

## Ethics Statement

Ethical approval for this study was secured from the Institutional Review Board (IRB) of ICDDR,B under Protocol Number PR‐22103. All procedures were performed in accordance with relevant guidelines and regulations. Informed written consent was obtained from all participants prior to data collection. To protect participant privacy, all data were fully anonymized by removing personal identifiers before analysis.

## Consent

All authors reviewed the manuscript thoroughly and provided consent for publication.

## Conflicts of Interest

Md Robed Amin and Maruf Ahmed Khan are affiliated with the Non‐communicable Disease Control (NCDC), Directorate General of Health Services (DGHS), Ministry of Health and Family Welfare (MoHFW), which provided funding for this study. All the remaining authors declare no conflicts of interest.

## Supporting Information

Additional supporting information can be found online in the Supporting Information section.

## Supporting information


**Supporting Information** In the supplementary materials, Figure S1: Map of four study sites, Figure S2: Patient flow and data collection points, Figure S3: Rader chart of mean responses to phq‐9 and gad‐7 items, Figure S4: Factor loading of GAD‐7 and PHQ‐9 items among male and female participants (*n* = 4,900), Figure S5: Factor loading of GAD‐7 and PHQ‐9 items by age group among the participants (*n* = 4,900), Table S1: Data collection tool/questionnaires, Table S2: Mean of PHQ‐9 and GAD‐7 items across demographic characteristics of respondent, Table S3: Chi‐square of the level of depressive symptoms and anxiety level, Table S4: Multivariate logistic regression of depressive and anxiety symptoms and their associated factors, Table S5: Network analysis matrix data (based on ggm visuals; cor: “polychoric correlation”), Table S6: Expected influence of the items (weighted sum of nodes), Table S7: Correlation stability analysis

## Data Availability

The anonymized datasets utilized in the present study are not accessible to the public as a precautionary measure to safeguard the confidentiality of participants. However, interested researchers may obtain access to these datasets upon making a reasonable request to the corresponding author.
