# Supplementary material for: Network and Factor Structure of Depression and Anxiety Symptoms in Telemental Healthcare Patients From Bangladesh: Evidence for Precision Mental Healthcare
Source: Depress Anxiety. 2026 Jun 17;2026:9552571. doi: 10.1155/da/9552571 (PMC13273640; doi:10.1155/da/9552571)
Supplement: Supplementary file 1 — Supporting Information In the supplementary materials, Figure S1: Map of four study sites, Figure S2: Patient flow and data collection points, Figure S3: Rader chart of mean responses to phq‐9 and gad‐7 items, Figure S4: Factor loading of GAD‐7 and PHQ‐9 items among male and female participants (n = 4,900), Figure S5: Factor loading of GAD‐7 and PHQ‐9 items by age group among the participants (n = 4,900), Table S1: Data collection tool/questionnaires, Table S2: Mean of PHQ‐9 and GAD‐7 items across demographic characteristics of respondent, Table S3: Chi‐square of the level of depressive symptoms and anxiety level, Table S4: Multivariate logistic regression of depressive and anxiety symptoms and their associated factors, Table S5: Network analysis matrix data (based on ggm visuals; cor: “polychoric correlation”), Table S6: Expected influence of the items (weighted sum of nodes), Table S7: Correlation stability analysis [file DA-2026-9552571-s001.pdf]

SUPPLEMENTARY MATERIALS

FIGURE S1: MAP OF FOUR STUDY SITES

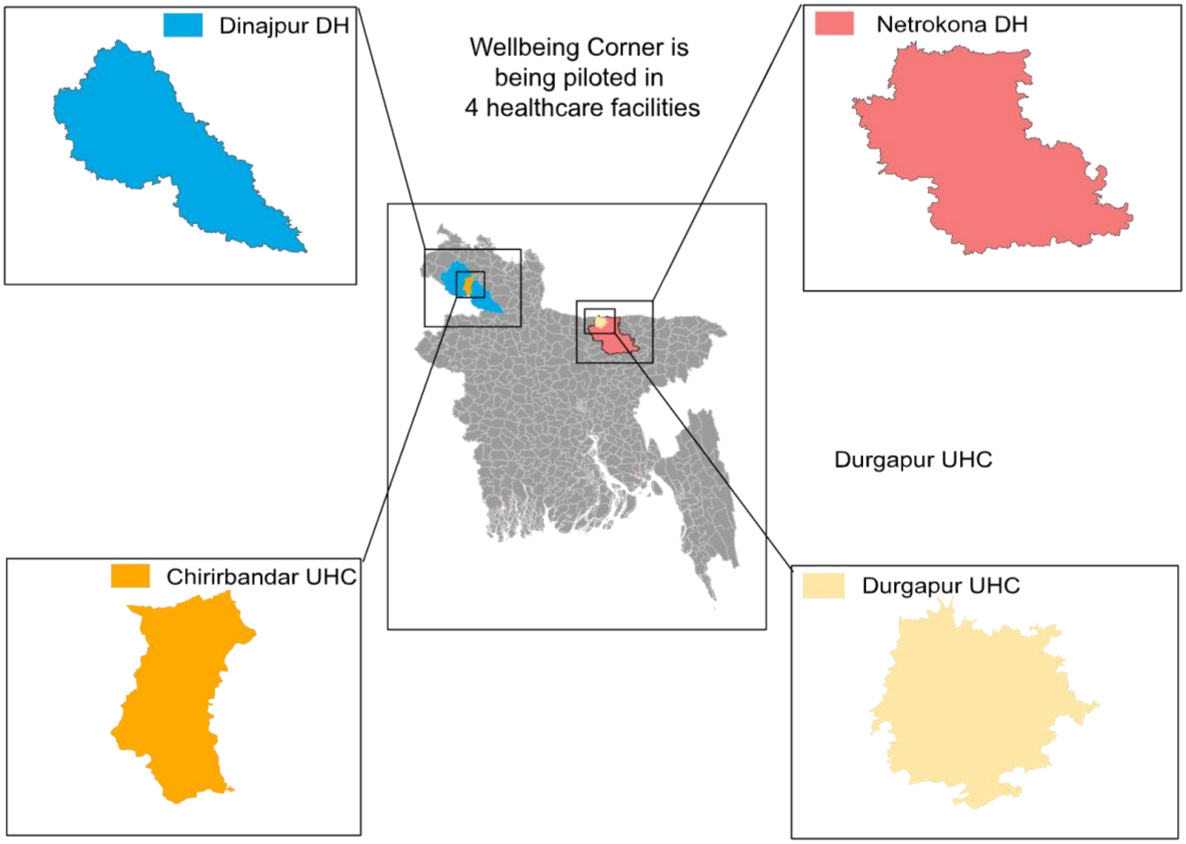

FIGURE S2: PATIENT FLOW AND DATA COLLECTION POINTS

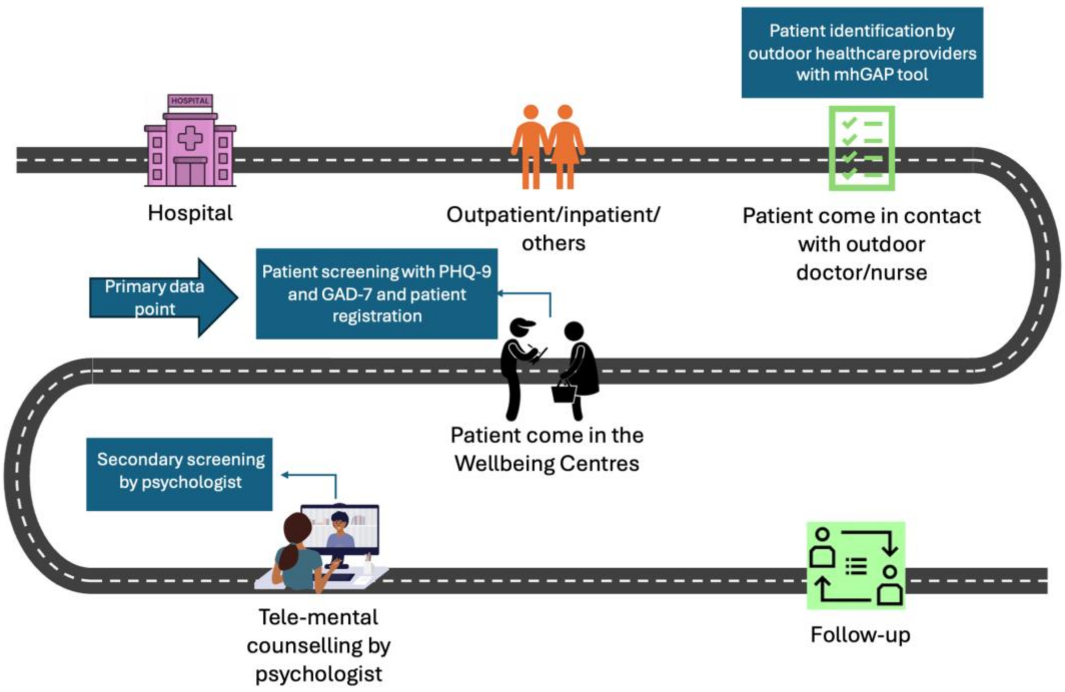

TABLE S1: EXAMPLE DATA COLLECTION TOOL/QUESTIONNAIRES

Participant's ID……………………………………………………………...........………………………….

Section A: Patient Health Questionnaires (PHQ-9)					
**SL**	**Variables**	**Not at all**	**Several days**	**More than half the days**	**Nearly every day**
A1	Little interest or pleasure in doing things	0	1	2	3
A2	Feeling down, depressed, or hopeless	0	1	2	3
A3	Trouble falling or staying asleep, or sleeping too much	0	1	2	3
A4	Feeling tired or having little energy	0	1	2	3
A5	Poor appetite or overeating	0	1	2	3
A6	Feeling bad about yourself — or that you are a failure or have let yourself or your family down	0	1	2	3
A7	Trouble concentrating on things, such as reading the newspaper or watching television	0	1	2	3
A8	Moving or speaking so slowly that other people could have noticed? Or the opposite — being so fidgety or restless that you have been moving	0	1	2	3

	around a lot more than usual				
A9	Thoughts that you would be better off dead or of hurting yourself in some way	0	1	2	3
		**Column totals**			
		**Total**			

**Section B: Generalised Anxiety Disorder (GAD-7)**					
**SL**	**Variables**	**Not at all**	**Several days**	**More than half the days**	**Nearly every day**
B1	Feeling nervous, anxious, or on edge	0	1	2	3
B2	Not being able to stop or control worrying	0	1	2	3
B3	Worrying too much about different things	0	1	2	3
B4	Trouble relaxing	0	1	2	3
B5	Being so restless that it is hard to sit still	0	1	2	3
B6	Becoming easily annoyed or irritable	0	1	2	3
B7	Feeling afraid, as if something awful might happen	0	1	2	3
		**Column totals**			
		**Total**			

SECTION C: DEMOGRAPHIC INFORMATION				
**SL**	**Characteristic**	**Select type**		**Skip**
D1	Age	___________________ (In year)		--> D2
D2	Religion	Islam	1	--> D3
		Hindu	2	
		Christian	3	
		Buddha	4	
		Others (specify) ______________	5	
D3	Current marital status	Married (or in a domestic partnership)	1	--> D4
		Divorced	2	
		Other (specify) ______________	3	
		Unmarried	4	
		Widow	5	
D4	Profession	Employee	1	--> D5
		Homemaker	2	
		Business	3	
		Agriculture	4	
		Unable to work due to disability	5	
		Other (Specify)	6	
		Student	7	
		Labourer/Worker	8	
		Unemployed	9	
D5	Total years of education	__________________________ (In year)		--> D6

D6	Monthly income	______________________________ (In Tk)	--> Next section

FIGURE S3: RADER CHART OF MEAN RESPONSES TO PHQ-9 AND GAD-7 ITEMS (N=4,900)

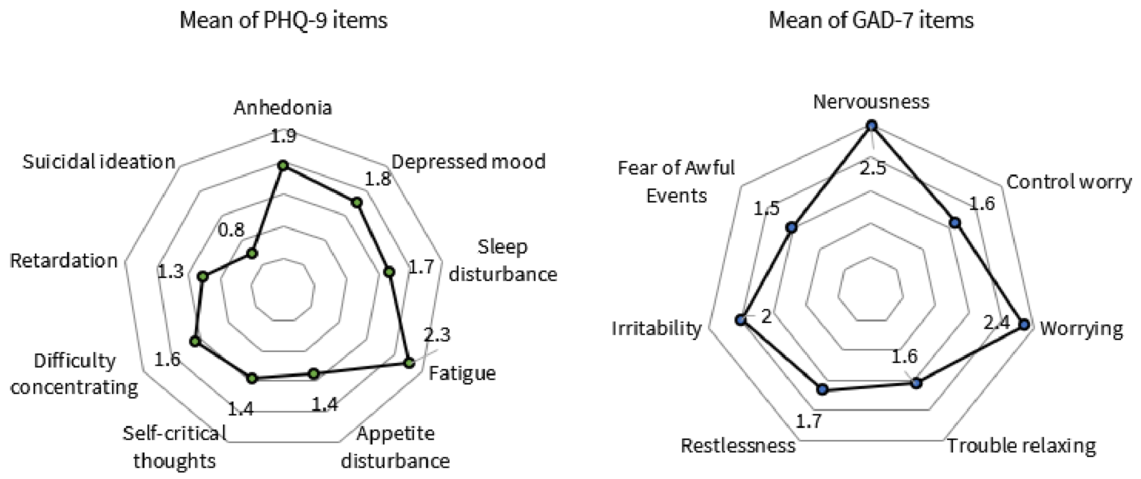

TABLE S2: MEAN OF PHQ-9 ABD GAD-7 ITEMS ACROSS DEMOGRAPHIC CHARACTERISTICS OF RESPONDENT (N=4,900)

PHQ-9

	Anhedonia	Depressed mood	Sleep problems	Low energy	Appetite change	Low self-esteem	Concentration difficulties	Retardation	Suicidal ideation
**Age, years**									
10-19	2.81	2.7	2.58	3.22	2.6	2.48	2.73	2.37	1.81
20-39	2.89	2.75	2.81	3.27	2.4	2.59	2.71	2.42	1.71
40-59	3.03	2.85	2.99	3.41	2.39	2.72	2.83	2.44	1.61
>=60	3	2.79	2.93	3.52	2.51	2.62	2.8	2.47	1.57
**Gender**									
Male	2.77	2.67	2.89	3.18	2.29	2.69	2.78	2.41	1.79
Female	2.96	2.79	2.8	3.35	2.49	2.58	2.74	2.42	1.66
**Religion**									
Muslim	2.92	2.76	2.82	3.32	2.45	2.6	2.73	2.4	1.69
Others[1]	2.88	2.76	2.86	3.18	2.29	2.72	2.87	2.64	1.8
**Marital status**									
Married	2.93	2.76	2.83	3.33	2.42	2.56	2.68	2.42	1.63
Unmarried	2.77	2.73	2.73	3.15	2.49	2.68	2.97	2.37	1.95
Divorced/widowed	3.18	3	3.02	3.57	2.51	2.96	2.95	2.63	1.72
**Profession**									
Involved in income generating activity[2]	2.75	2.67	2.91	3.17	2.26	2.68	2.68	2.38	1.79
Homemaker	2.99	2.79	2.81	3.38	2.49	2.57	2.69	2.42	1.62
Student	2.77	2.72	2.69	3.16	2.5	2.6	2.96	2.38	1.86
Unemployed	3.06	2.87	3.07	3.47	2.3	2.88	3.1	2.7	1.85
**Education, years**									
0-5	2.98	2.79	2.86	3.38	2.51	2.66	2.73	2.36	1.68
6-10	2.94	2.77	2.78	3.32	2.45	2.56	2.7	2.47	1.69
>=11	2.78	2.72	2.83	3.2	2.32	2.6	2.84	2.43	1.72
**Household income, taka per month**									
Very low	2.95	2.83	2.89	3.31	2.49	2.69	2.76	2.56	1.8

Lower middle	2.95	2.78	2.8	3.35	2.48	2.62	2.72	2.42	1.67
Middle	2.85	2.67	2.9	3.27	2.44	2.55	2.75	2.34	1.7
High	2.81	2.73	2.72	3.17	2.15	2.5	2.83	2.35	1.68
**Patient is from**									
Referred from outdoors[3]	2.9	2.72	2.94	3.44	2.5	2.68	2.81	2.4	1.57
Referred by other hospital[4]	2.75	2.75	3.35	3.51	2.75	2.79	2.94	2.76	1.71
Walk-in	3.42	2.98	3.41	3.52	2.53	2.71	2.92	2.96	1.42
**Region**									
Sub-district	3.06	2.84	2.59	3.19	2.29	2.43	2.62	2.45	1.45
District	2.78	2.69	3.03	3.43	2.58	2.77	2.86	2.39	1.93

[1] *Hindu, Christian*
[2] *Job, business, farmer, mason, or daily labour.*
[3] *gynaecology, medicine indoor, medicine outdoor, or emergency.*
[4] *community clinic, other hospitals*

GAD-7

	**Nervousness**	**Unable to control worry**	**Worrying**	**Trouble relaxing**	**Restlessness**	**Irritability**	**Fear off awful events**
**Age, years**							
10-19	3.3	2.62	3.09	2.51	2.78	3.14	2.58
20-39	3.54	2.73	3.46	2.62	2.81	3.02	2.51
40-59	3.66	2.81	3.57	2.82	3.01	2.93	2.4
>=60	3.71	2.74	3.55	2.82	3.06	2.77	2.43
**Gender**							
Male	3.56	2.89	3.36	2.68	2.86	3.04	2.43
Female	3.54	2.68	3.45	2.65	2.87	2.99	2.51
**Religion**							
Muslim	3.55	2.72	3.42	2.66	2.87	3.01	2.49
Others[1]	3.48	2.82	3.49	2.62	2.79	2.93	2.55
**Marital status**							
Married	3.55	2.69	3.46	2.67	2.87	2.97	2.48
Unmarried	3.43	2.82	3.2	2.57	2.78	3.16	2.59

Divorced/widowed	3.76	2.97	3.73	2.92	3.11	2.92	2.38
**Profession**							
Involved in income generating activity[2]	3.59	2.83	3.43	2.69	2.83	3.06	2.4
Homemaker	3.55	2.66	3.47	2.67	2.89	2.95	2.49
Student	3.41	2.78	3.24	2.53	2.72	3.21	2.6
Unemployed	3.66	3.09	3.46	2.89	3.17	2.9	2.57
**Education, years**							
0-5	3.6	2.69	3.43	2.72	3	2.89	2.45
6-10	3.49	2.73	3.4	2.67	2.86	3.07	2.54
>=11	3.52	2.77	3.47	2.55	2.69	3.07	2.49
**Household income, taka per month**							
Very low	3.49	2.85	3.48	2.65	2.85	2.99	2.56
Lower middle	3.54	2.69	3.44	2.69	2.89	2.98	2.5
Middle	3.56	2.74	3.35	2.65	2.88	3.05	2.45
High	3.54	2.71	3.42	2.54	2.74	3.11	2.4
**Patient is from**							
Referred by hospital indoor & outdoor[3]	3.61	2.75	3.49	2.79	3	3	2.37
Referred by other hospital[4]	3.75	3.27	3.43	2.9	3.19	3.25	2.73
Walk-in	3.7	2.89	3.84	2.69	2.66	3.41	2.48
**Region**							
Sub-district	3.54	2.37	3.57	2.38	2.69	2.83	2.47
District	3.54	3.06	3.3	2.93	3.03	3.17	2.52

[1] *Hindu, Christian*
[2] *Job, business, farmer, mason, or daily labour.*
[3] *gynaecology, medicine indoor, medicine outdoor, or emergency.*
[4] *community clinic, other hospital*

TABLE S3: PERCENTAGE DISTRIBUTION AND CHI-SQUARE ASSOCIATION OF DEPRESSION AND ANXIETY SYMPTOM SEVERITY ACROSS RESPONDENT DEMOGRAPHICS (N=4,900)

Depressive symptoms

Variables		None	Mild	Moderate	Moderately sever	Severe	P-value
**Age, years**	Age 10-19, years	1	16	35	32	16	-
	Age 20-39, years	1	16	33	34	17	
	Age 40-59, years	1	12	28	40	18	
	Age >=60, years	0	11	33	38	18	
**Gender**	Male	1	17	32	34	16	0.050
	Female	1	14	33	36	17	
**Religion**	Muslim	1	15	33	35	17	0.8
	Other religion	1	13	32	37	18	
**Marital status**	Married	1	15	33	35	16	-
	Unmarried	1	16	31	34	19	
	Divorced/widowed	0	4	27	43	26	
**Profession**	Generate Income	2	16	33	33	15	-
	Homemaker	1	14	33	36	17	
	Student	1	16	31	35	18	
	Unemployed	0	10	26	36	28	
**Education, years**	Education 0-5, years	0	13	32	37	18	-
	Education 6-10, years	1	14	34	34	17	
	Education >=11, years	1	17	31	34	17	
**Household income, taka per month**	Very low	1	14	29	33	23	<0.001
	Lower middle	1	13	33	37	16	
	Middle	2	16	32	34	17	
	High	1	21	34	28	16	
Referred from	Referred from outdoors	0	13	32	40	16	-
	Referred by other hospital	0	11	16	51	22	
	Walk-in	0	2	22	53	23	
**Catchment area**	Region, sub-district	1	14	40	35	10	<0.001
	Region, district	1	15	25	35	24	

(Maximum N = 4,900; sample size varies by variable due to missing covariate data)

Anxiety symptoms

Variables		None	Mild	Moderate	Severe	P-value
**Age, years**	Age 10-19, years	1	20	41	37	-
	Age 20-39, years	1	15	42	43	
	Age 40-59, years	0	11	39	50	
	Age >=60, years	1	11	41	48	
**Gender**	Male	1	16	36	48	<0.001
	Female	1	14	43	43	
**Religion**	Muslim	1	15	41	44	0.8
	Other religion	1	15	42	43	
**Marital status**	Married	1	14	42	43	-
	Unmarried	1	19	36	44	
	Divorced/widowed	0	6	38	56	
**Profession**	Generate Income	1	14	39	46	-
	Homemaker	1	14	43	42	
	Student	1	19	37	43	
	Unemployed	0	13	28	60	
**Education, years**	Education 0-5, years	0	14	41	45	-
	Education 6-10, years	1	14	41	44	
	Education >=11, years	1	17	40	43	
**Household income, taka per month**	Very low	1	13	39	47	<0.001
	Lower middle	1	14	42	44	
	Middle	1	16	41	42	
	High	1	19	39	42	
Referred from	Referred from outdoors	0	12	42	46	-
	Referred by other hospital	0	6	25	68	
	Walk-in	0	5	39	56	
**Catchment area**	Region, sub-district	0	15	54	31	-
	Region, district	1	15	28	56	

(Maximum N = 4,900; sample size varies by variable due to missing covariate data)

TABLE S4: MULTIVARIATE LOGISTIC REGRESSION OF DEPRESSIVE AND ANXIETY SYMPTOMS AND THEIR ASSOCIATED FACTORS

Model for Depressive symptoms

**Characteristic**	**aOR**[1]	**95% CI**[1]	**p-value**
Age, years			
10-19	—	—	
20-40	1.12	0.87, 1.44	0.4
41-91	1.48	1.08, 2.03	0.016
Gender			
Female	—	—	
Male	0.67	0.50, 0.88	0.005
Religion			
Muslim	—	—	
Others	1.20	0.89, 1.64	0.2
Marital status			
Married	—	—	
Others	3.16	1.82, 6.04	<0.001
Unmarried	1.20	0.85, 1.71	0.3
Profession			
Generate income	—	—	
Homemaker	0.87	0.63, 1.17	0.4
Student	1.00	0.68, 1.46	>0.9
Unemployed	1.59	0.97, 2.71	0.075
Education, years			
0-5	—	—	

Characteristic	aOR[1]	95% CI[1]	p-value
6-10	1.05	0.86, 1.28	0.7
>=11	0.87	0.70, 1.09	0.2
Referred from			
Referred from outdoors	—	—	
Referred by other hospital	1.21	0.91, 1.53	0.18
Walk-in	0.93	0.71, 1.25	0.3
Income, taka			
<10000	—	—	
10000-19999	1.21	0.95, 1.53	0.12
20000-29999	0.94	0.71, 1.25	0.7
>=30000	0.73	0.54, 0.99	0.044

[1] OR = Odds Ratio, CI = Confidence Interval

Model for Anxiety symptoms

Characteristic	OR[1]	95% CI[1]	p-value
Age, years			
10-19	—	—	
20-40	1.53	1.21, 1.93	<0.001
41-91	2.14	1.57, 2.92	<0.001
Gender			
Female	—	—	
Male	0.79	0.60, 1.04	0.092
Religion			
Muslim	—	—	
Others	1.03	0.78, 1.39	0.8
Marital status			

Characteristic	OR[1]	95% CI[1]	p-value
Married	—	—	
Others	2.05	1.27, 3.54	0.006
Unmarried	0.91	0.64, 1.28	0.6
Profession			
Generate income	—	—	
Homemaker	0.82	0.60, 1.11	0.2
Student	0.96	0.66, 1.40	0.8
Unemployed	1.20	0.75, 1.97	0.5
Education, years			
0-5	—	—	
6-10	1.22	1.00, 1.49	0.048
>=11	1.00	0.80, 1.25	>0.9
Referred from			
Referred from outdoors	—	—	
Referred by other hospital	1.3	0.9, 1.49	0.2
Walk-in	0.89	0.72, 1.21	0.6
Income, taka			
<10000	—	—	
10000-19999	1.09	0.86, 1.38	0.5
20000-29999	0.94	0.71, 1.25	0.7
>=30000	0.81	0.59, 1.10	0.2

[1] OR = Odds Ratio, CI = Confidence Interval

FIGURE S4: FACTOR LOADING OF GAD-7 AND PHQ-9 ITEMS AMONG MALE AND FEMALE PARTICIPANTS (N=4,900).

For both genders, "depressed mood" (λ=0.62 for males and λ=0.56 for females) and "anhedonia" (λ=0.55 for males and λ=0.5 for females) are the strongest indicators of depression. In terms of anxiety, "control worry" (λ=0.69 for males and λ=0.68 for females) and "nervousness" (λ=0.7 for males and λ=0.59 for females) are the most strongly associated symptoms for both genders.

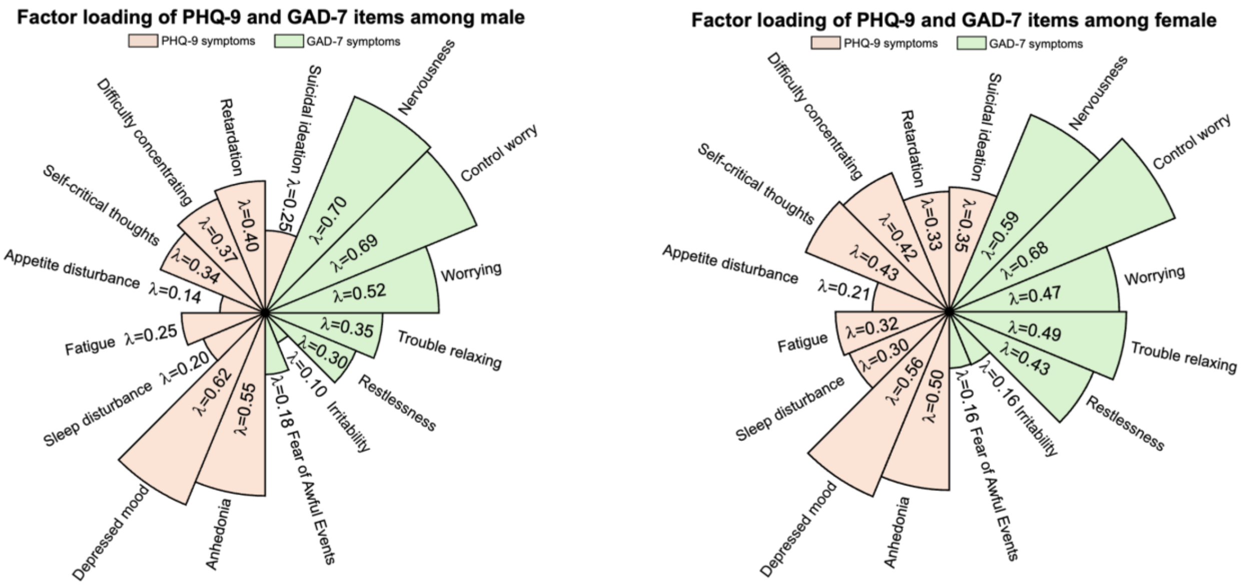

Figure S5: Factor loading of GAD-7 and PHQ-9 items by age group among the participants (n=4,900

For all age groups, "depressed mood" and "anhedonia" were the strongest indicators of depression. In terms of anxiety, "control worry" and "nervousness" were the most strongly associated symptoms for all age groups of the participants.

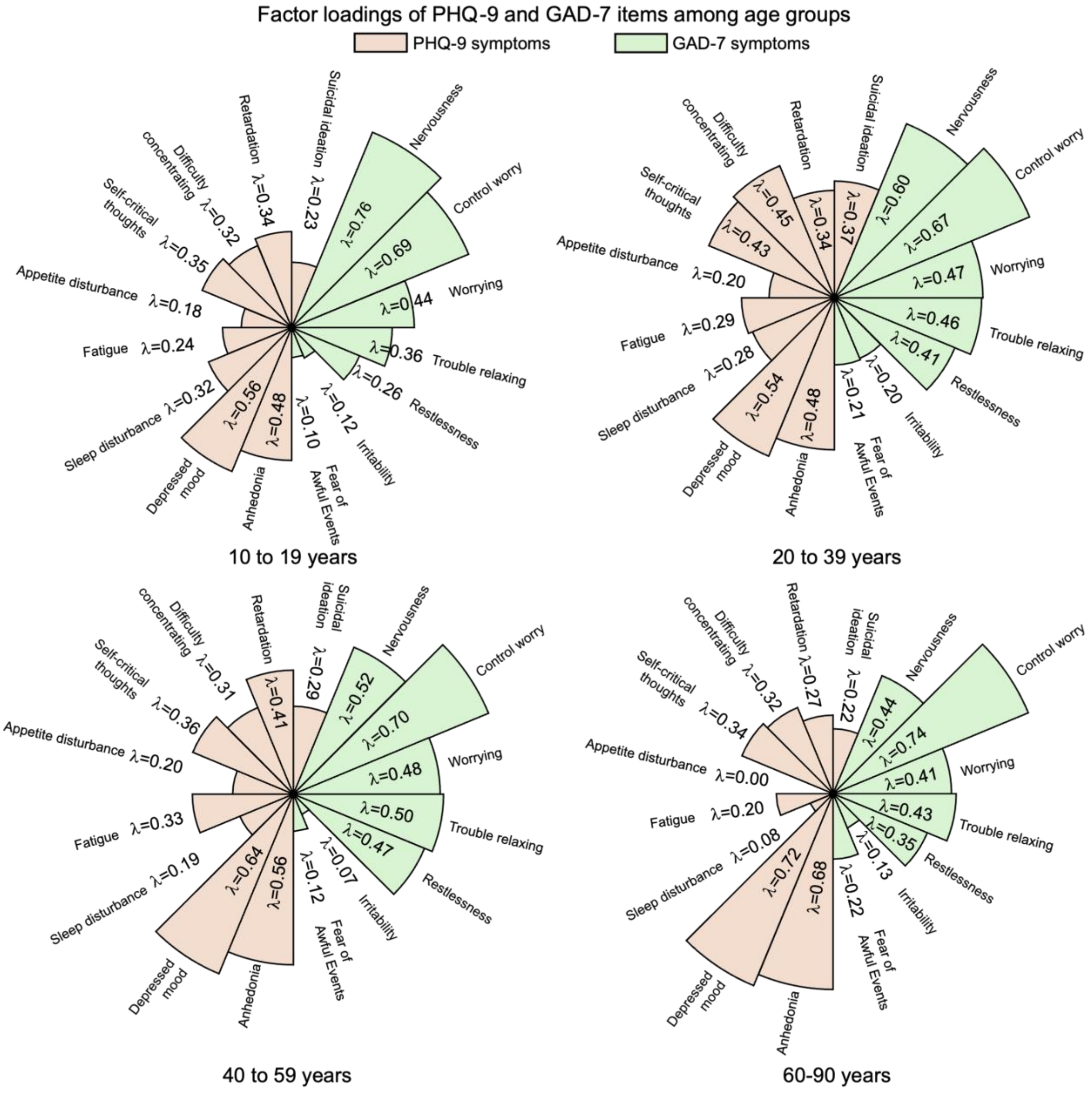

TABLE S5: NETWORK ANALYSIS MATRIX DATA (BASED ON GGM VISUALS; COR: "POLYCHORIC CORRELATION")

	PHQ 1	PHQ 2	PHQ 3	PHQ 4	PHQ 5	PHQ 6	PHQ 7	PHQ 8	PHQ 9	GAD 1	GAD 2	GAD 3	GAD 4	GAD 5	GAD 6
PHQ 2	0.61														
PHQ 3	0.09	0.11													
PHQ 4	0.17	0.13	0.24												
PHQ 5	0.06	0.05	0.26	0.33											
PHQ 6	0.16	0.25	0.13	0.14	0.06										
PHQ 7	0.22	0.25	0.18	0.23	0.10	0.24									
PHQ 8	0.25	0.27	0.16	0.17	0.03	0.15	0.18								
PHQ 9	0.13	0.19	0.09	0.03	0.10	0.51	0.15	0.14							
GAD 1	0.25	0.25	0.11	0.23	0.04	0.37	0.23	0.09	0.23						
GAD 2	0.09	0.24	0.28	0.27	0.16	0.37	0.31	0.21	0.31	0.64					
GAD 3	0.27	0.31	0.22	0.22	0.05	0.27	0.21	0.27	0.08	0.65	0.46				
GAD 4	0.19	0.22	0.44	0.30	0.23	0.26	0.26	0.23	0.16	0.22	0.34	0.19			
GAD 5	0.15	0.17	0.27	0.34	0.25	0.23	0.28	0.12	0.16	0.21	0.29	0.12	0.65		

GAD 6	0.08	0.15	0.16	0.14	0.11	0.22	0.20	0.13	0.27	0.01	0.20	0.10	0.16	0.13	
GAD 7	0.13	0.20	0.00	0.09	0.03	0.19	0.14	0.15	0.19	0.12	0.19	0.09	0.14	0.11	-0.01

TABLE S6. EXPECTED INFLUENCE OF THE ITEMS (WEIGHTED SUM OF NODES)

	EI Values
GAD2	1.760476
GAD4	1.270112
GAD1	0.799531
PHQ6	0.69857
GAD3	0.636321
GAD5	0.58612
PHQ2	0.485741
PHQ7	0.203857
PHQ4	-0.03306
PHQ1	-0.27059
PHQ9	-0.39024
PHQ3	-0.42333
PHQ8	-0.67464
GAD6	-1.34461
PHQ5	-1.59573
GAD7	-1.70853

TABLE S7: CORRELATION STABILITY ANALYSIS USING CASE-DROPPING BOOTSTRAP.

NPerson indicates the number of observations retained in each subsample. *Drop%* indicates the proportion of cases removed relative to the full sample. *n* indicates the number of bootstrap samples at each sampling level. The central stability (CS) coefficient represents the maximum proportion of cases that can be dropped while maintaining a correlation of at least 0.7 between centrality estimates from the original sample and subset networks.

Sampling levels tested:

	NPerson	Drop%	n
1	1225	75	93
2	1606	67.2	120
3	1987	59.4	87
4	2368	51.7	115
5	2749	43.9	94
6	3131	36.1	99
7	3512	28.3	92
8	3893	20.6	103
9	4274	12.8	101
10	4655	5	96

CS Coefficients:

• betweenness: 0.283 (caseMin = 0.206, caseMax = 0.361)

• closeness: 0.75 (caseMin = 0.672, caseMax = 1)

• expectedInfluence: 0.75 (caseMin = 0.672, caseMax = 1)

• strength: 0.75 (caseMin = 0.672, caseMax = 1)
